# Glial Cell Lineage Expression of Mutant Ataxin-1 and Huntingtin Induces Developmental and Late-Onset Neuronal Pathologies in Drosophila Models

**DOI:** 10.1371/journal.pone.0004262

**Published:** 2009-01-23

**Authors:** Takuya Tamura, Masaki Sone, Mayumi Yamashita, Erich E. Wanker, Hitoshi Okazawa

**Affiliations:** 1 Department of Neuropathology, Medical Research Institute, Tokyo Medical and Dental University, Tokyo, Japan; 2 Medical Top Track Program, Medical Research Institute, Tokyo Medical and Dental University, Tokyo, Japan; 3 Department of Neurogenetics, Max-Delbrück Center for Molecular Medicine, Berlin-Buch, Germany; University of Cambridge, United Kingdom

## Abstract

**Background:**

In several neurodegenerative disorders, toxic effects of glial cells on neurons are implicated. However the generality of the non-cell autonomous pathologies derived from glial cells has not been established, and the specificity among different neurodegenerative disorders remains unknown.

**Methodology/Principal Findings:**

We newly generated Drosophila models expressing human mutant huntingtin (hHtt103Q) or ataxin-1 (hAtx1-82Q) in the glial cell lineage at different stages of differentiation, and analyzed their morphological and behavioral phenotypes. To express hHtt103Q and hAtx1-82Q, we used 2 different Gal4 drivers, gcm-Gal4 and repo-Gal4. Gcm-Gal4 is known to be a neuroglioblast/glioblast-specific driver whose effect is limited to development. Repo-Gal4 is known to be a pan-glial driver and the expression starts at glioblasts and continues after terminal differentiation. Gcm-Gal4-induced hHtt103Q was more toxic than repo-Gal4-induced hHtt103Q from the aspects of development, locomotive activity and survival of flies. When hAtx1-82Q was expressed by gcm- or repo-Gal4 driver, no fly became adult. Interestingly, the head and brain sizes were markedly reduced in a part of pupae expressing hAtx1-82Q under the control of gcm-Gal4, and these pupae showed extreme destruction of the brain structure. The other pupae expressing hAtx1-82Q also showed brain shrinkage and abnormal connections of neurons. These results suggested that expression of polyQ proteins in neuroglioblasts provided a remarkable effect on the developmental and adult brains, and that glial cell lineage expression of hAtx1-82Q was more toxic than that of hHtt103Q in our assays.

**Conclusion/Significance:**

All these studies suggested that the non-cell autonomous effect of glial cells might be a common pathology shared by multiple neurodegenerative disorders. In addition, the fly models would be available for analyzing molecular pathologies and developing novel therapeutics against the non-cell autonomous polyQ pathology. In conclusion, our novel fly models have extended the non-cell autonomous pathology hypothesis as well as the developmental effect hypothesis to multiple polyQ diseases. The two pathologies might be generally shared in neurodegeneration.

## Introduction

Effects of glial expression of mutant proteins (or non-cell autonomous effects) on the pathology have been suggested in amyotrophic lateral sclerosis (ALS) and in spinocerebellar ataxia type 7 (SCA7). Primary mouse spinal motor neurons expressing mutant superoxide dismutase 1 (SOD1) do not provoke motor neuron degeneration, whereas when motor neurons were generated from embryonic stem cells (ESCs), co-culture with primary glial cells from transgenic mice expressing mutant SOD1 induces neurodegenerative changes of co-cultured neurons [1], [2]. Furthermore, conditioned media by astrocytes but not fibroblasts, microglias, or cortical neurons induces cell death of motor neurons, suggesting soluble factor(s) mediate the non-cell autonomous effect. These effects *in vitro* were confirmed also *in vivo*. Selective Cre-mediated gene excision of mutant SOD1 in astrocytes improved the survival of SOD1 expressing mice [3], while mutant SOD1 expression in cell types other than motor neurons and oligodendrocytes seem to accelerate the onset of motor neuron disease phenotype [4]. Specifically in the case of mutant SOD1 transgenic mice, secreted mutant SOD1 protein from reactive astrocytes might be a mediator of the glial toxicity [5].

In polyglutamine (polyQ) diseases, the La Spada group reported that mutant ataxin-7 in Bergmann glias induces ataxia and neurodegeneration of Purkinje cells in mice [6]. The Orr group also reported that embryonic expression of human ataxin-1 (hAtx1), the causative gene product of SCA1, which interacts with retinoic acid orphan receptor alpha (ROR-α) and disturbs Purkinje cell development, affects the pathology in adulthood [7]. It indicates that the mutant gene expression and its interaction with mediator molecules during development are critical factors for the SCA1 pathology. All these studies suggested that the non-cell autonomous effect of glial cells might be a common pathology shared by multiple neurodegenerative disorders.

In this study, to test this hypothesis of non-cell autonomous effects by glial cells as well as by stem/progenitor cells, we generated and analyzed phenotypes of Drosophila models expressing human mutant Htt or Atx1 in the glial cell lineage at different stages of differentiation. In both cases, severe phenotypes are observed in development, locomotive activity and survival of flies. Morphological analyses of these flies revealed severe degeneration of neurons in the brain. These results further support the concept that glial cells expressing mutant proteins provides a non-cell autonomous effect on neurons. In addition, the fly models would be available for analyzing molecular pathologies and developing novel therapeutics against the non-cell autonomous polyQ pathology.

## Results

### Generation of fly models expressing mutant polyQ proteins in glial cell lineage

**Figure 1:**
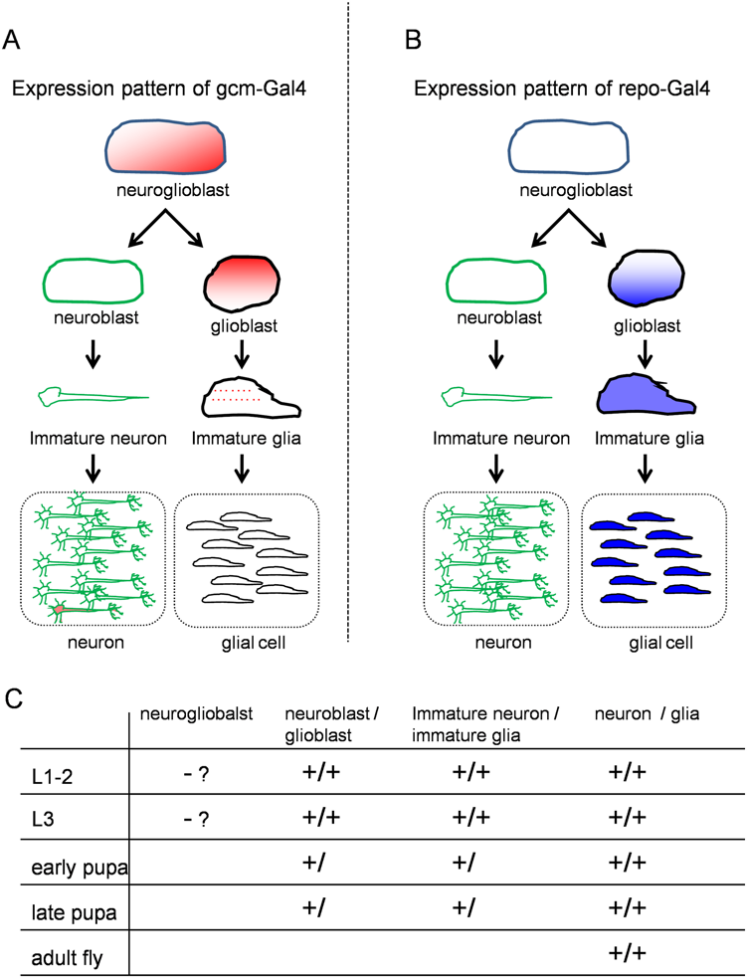
Expression patterns of *gcm* and *repo* genes during glial cell lineage differentiation. (A) *Gcm* is expressed in neuroglioblasts, glioblasts, and transiently in immature glia cells [8], [24]. Exceptionally, expression of gcm is observed in a very few number of interneurons in the ventral nerve cord from L1 to adult stage [9]. (B) A scheme of the expression pattern of *repo*. In addition, Repo is detected in longitudinal glioblasts and glial cells [25]. (C) Existence of different types of glial cell lineage and neuronal cell lineage cells at developmental stages.

We used two Gal4 drivers, *repo-Gal4* and *gcm-Gal4* to express polyglutamine proteins in glial lineage cells. Glial Cell Missing (*gcm*) is a transcription factor that regulates glial cell lineage commitment to longitudinal glioblast cells [8]. After the commitment, *gcm* expression decreases rapidly but sustains until immature glial cells [8] (Fig. 1A). Exceptionally, a small percentage of mature neurons in ventral nerve cord seem to express *gcm* [9] beside glial cell lineage. Reversed Polarity (*repo*), a homeobox gene required for the differentiation and maintenance of glia function, is another marker of glial cell lineage [10]. Repo is expressed from glioblasts, immature to mature glial cells but not in neuroglioblasts (Fig. 1B). All glial cells except midline glias express *repo* [8], [10]. GCM protein is known to regulate transcription of *repo* in glial cells [11], [12].

In Figure 1, such differences between *gcm* and *repo* expression patterns are summarized. *Gcm* is mainly expressed around commitment to glial cell lineage. Expression level of *repo* is from immature to mature glial cells is far higher than that of *gcm*. Therefore, in brief, we can induce glial cell-specific gene expression by *repo* driver and stem/progenitor cell-specific gene expression by *gcm* driver in the glial cell lineage (Fig 1A, B). Existence of different types of cells in glial cell lineage or neuronal cell lineage is also summarized in Table (Fig. 1C).

### Behavioral and survival effects of glial cell-lineage expression of mutant huntingtin

**Figure 2:**
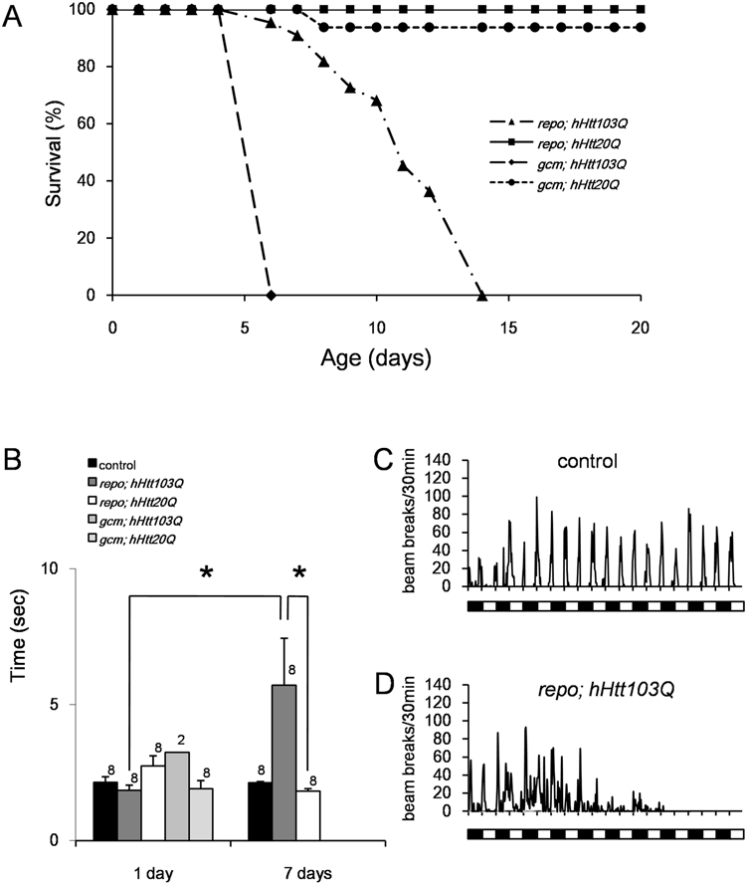
Effects of glial expressions of hHtt103Q on survival, locomotive and spontaneous activities of adult flies. (A) The survival curves of adult flies show that the flies expressing hHtt103Q with gcm-Gal4 driver died by Day 6 and the flies expressing hHtt103Q with repo-Gal4 driver died by Day 14. (B) Negative geotactic responses of hHtt103Q or hHtt20Q expressing flies. Because *gcm; hHtt103Q* flies died within 1 week as shown in A, we could not test them at 7 days. Asterisk: p<0.05 in Student's *t-test*. The mean+SE and the number of flies used for each experiment are shown. (C) and (D) indicate spontaneous activity at Day 2 of control and *repo; hHtt103Q* flies, respectively. Open and filled boxes under the graph indicate Light and Dark cycle (L∶D = 12 hr∶12 hr). We could not test *gcm; hHtt103Q* flies due to the low birth rate.

We crossed male *repo-Gal4* flies balanced by a balancer chromosome (*TM3*, *sb*) with *UAS-hHtt103Q* virgin females because homo *repo-Gal4* fly is lethal. The number of F1 flies was not different between *repo-Gal4* positive and negative flies (Table 1), suggesting that embryogenesis of the flies expressing mutant human Htt in glial cells was largely normal. Therefore, we tested lifespan and motor activity of adult *repo-Gal4/ UAS-hHtt103Q* (*repo; hHtt103Q*) flies, and found lifespan shortening and abnormal motor activities (Fig. 2A, B). We also observed spontaneous activity of the flies, which was basically in accordance with the Light-Dark cycle but partially arrhythmic (Fig. 2C vs 2D). In addition, their spontaneous activities decreased gradually during the test period (Fig. 2D).

**Table 1:** The number of adult flies which expressing hHtt103Q induced by gcm-Gal4 is reduced.

driver	hHtt103Q expression	# of flies born
gcm-Gal4	+	3
	−	120
repo-Gal4	+	60
	−	60

We next employed *gcm-Gal4* driver for expression of mutant Htt. Because the homo *gcm-Gal4* flies were lethal, *gcm-Gal4* flies were balanced by a balancer chromosome (*Gla*). Then we crossed them with virgin female *UAS-hHtt103* flies like generation from *repo-Gal4* flies. In this case, the birth rate of *gcm-Gal4/+*; *UAS-hHtt103Q/+* (*gcm; hHtt103Q*) fly was remarkably reduced (Table 1), suggesting that developmental abnormalities were induced by mutant Htt expression in neuroglioblasts/glioblasts. Although the number of the *gcm; hHtt103Q* adult flies were small, all the flies were available for behavioral analyses. We found their lifespan to be shortened remarkably (Fig. 2A). Their anti-gravity climbing activity was not impaired within 24 hrs after eclosion, but they became unable to move within few days (Fig. 2B). Because of the small number of adult flies and their very short lifespan we could not calculate their daily activities.

### Pathological effects of glial cell-lineage expression of mutant huntingtin

**Figure 3:**
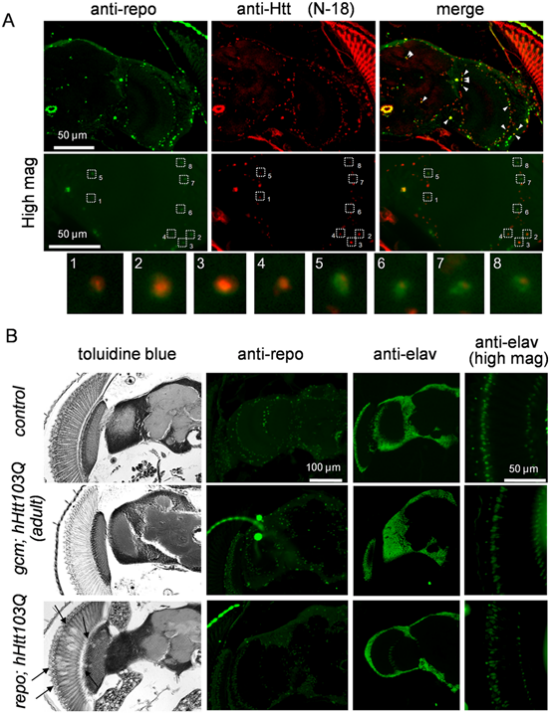
hHtt103Q expression in glial cell lineage induces pathological change in the central nervous system. (A) In *repo; hHtt103Q* flies, mutant Htt stained by anti-hHtt N-18 antibody was clearly colocalized with a glial cell marker, repo stained by anti-repo antibody (upper panels, arrow heads). High magnification shows a negative relationship between the expression level of hHtt103Q and that of repo (middle panels, dotted squares numbered). Glial cells marked with squares in middle panels are magnified in lower panels. Four examples of high-Htt103Q cells (1∼4) and 4 examples of high-repo cells (5∼8) are shown. (B) Morphological changes of brains in the *gcm; hHtt103Q* and *repo; hHtt103Q* flies. The coronal sections of the head were stained with toluidine blue, anti-repo antibody (glial cell marker), and anti-elav antibody (pan-neuron marker). Arrows in *repo; hHtt103Q* flies indicate vacuolar changes.

We next performed morphological analysis of these fly models. Expression of human mutant Htt protein with 103 polyQ repeats (hHtt103Q) was confirmed by immunohistochemistry with N-18 anti-htt antibody (Fig. 3A, N-18). The distribution pattern of the mutant hHtt inclusion bodies in the brain at Day 2 was consistent with that of glial cells (Fig. 3A, repo). We also performed double staining of repo and mutant hHtt and the validity of the *repo-Gal4* driver was confirmed (Fig. 3A, merge arrow heads and 1∼8).

Toluidine blue staining revealed vacuolar changes in retina and lamina of the *repo; hHtt103Q* flies (Fig. 3B, arrows), but not in the *gcm; hHtt103Q* flies. The number or signal intensity of glial cells stained by anti-repo antibody were reduced and photoreceptor cells stained by anti-elav antibody showed abnormal alignment and morphology at a high magnification in the retina of the *repo; hHtt103Q* flies (Fig. 3B). In the *gcm; hHtt103Q* flies, morphological change was not remarkable (Fig. 3B).

### Developmental effects of glial cell-lineage expression of mutant ataxin-1

No adult fly was obtained in expression of mutant human ataxin-1 (hAtx1-82Q) under the control of *gcm-Gal4* or *repo-Gal4* driver. Therefore, we tried to determine the stage when the development of the expressers was inhibited. To discriminate the flies expressing hAtx1-82Q, we employed GFP-balancers. As expected, we could distinguish *hAtx1-82Q/+ (or Y); gcm-Gal4/+* flies from *hAtx1-82Q/+ (or Y); CyO,GFP/+* flies by fluorescence even at larval stage. *hAtx1-82Q/+ (or Y);;repo-Gal4/+* and *hAtx1-82Q /+ (or Y);;TM3, GFP sb/+* could be also distinguished by GFP.

**Figure 4:**
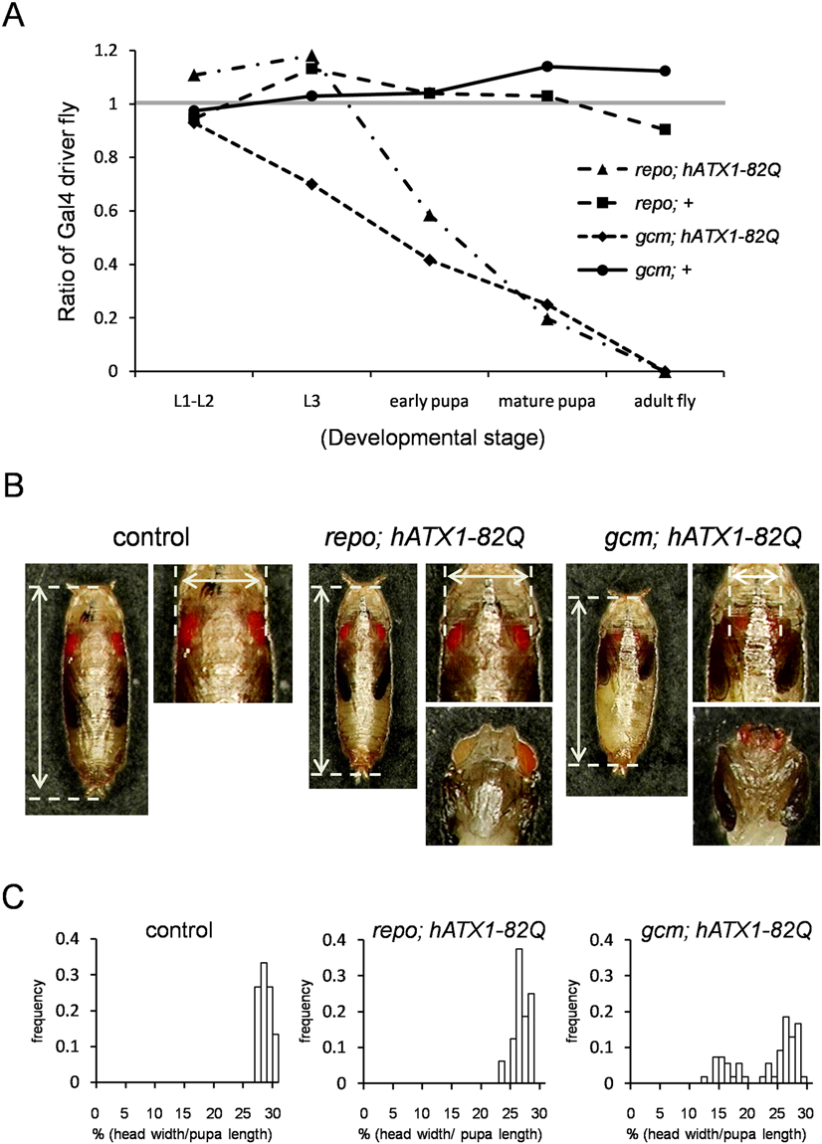
Glial cell lineage expression of hAtx1-82Q induces abnormal development. (A) The graph shows survival from larvae to adult fly of the hAtx1-82Q expressing (*repo; hATX1-82Q* and *gcm; hATX1-82Q*) or non-expressing siblings. The *repo; hATX1-82Q* and *gcm; hATX1-82Q larvae* (F1) are generated by crossing *UAS-hATX1-82Q* virgin females and *repo-Gal4/TM3, GFP Sb* or *gcm-Gal4/CyO*, *GFP* males. repo+ and gcm+ are F1 of *WT* virgin females and *repo-Gal4/TM3*, *GFP Sb* or *gcm-Gal4/CyO*, *GFP* males. The ratio of GFP(−)/GFP(+) larvae was calculated. The gray line in the graph is the ideal ratio for no toxicity. (B) Head size in *gcm; hATX1-82Q* at mature pupa stage was evaluated. Vertical and horizontal arrows indicate the length of pupae and the width of their heads, respectively. Wild type (control), *repo; hATX1-82Q* F1 and *gcm; hATX1-82Q* F1-pupae (GFP-negative) were examined. The lower panels show the heads after removing the capsule. (C) Histograms show the head width /pupa length ratio (%) of pupae. The frequency of ratio was plotted in each histogram, and the numbers of examined control, *repo; hATX1-82Q* and *gcm; hATX1-82Q* were 15, 16 and 54, respectively.

At 1st and 2nd instar larvae (L1–L2), hAtx1-82Q driven by *gcm-* and *repo-Gal4* did not affect the survival (Fig. 4A). Afterwards the ratio of F1 flies expressing hAtx1-82Q was declined during development. The initial effect of hAtx1-82Q was detected at 3rd instar larvae (L3) in *gcm-Gal4* driver (Fig. 4A) and at early pupa with *repo-Gal4* driver (Fig. 4A). A part of larvae expressing hAtx1-82Q by *gcm-* or *repo-Gal4* became pupa but not adult flies (Fig. 4A). The toxicity of hAtx1-82Q expression may be variable from larva to eclosion stage, stochastically.

Interestingly we found a part of pupae of *hAtx1-82Q /+ (or Y); gcm-Gal4/+ (gcm; hAtx1-82Q)* to have remarkably small heads (Fig. 4B). To quantitatively evaluate the small head phenotype, the ratio between head width and length of pupa was calculated (Fig. 4B and C). The histogram of the ratio in *gcm; hAtx1-82Q* Q flies was clearly biphasic (Fig. 4C), and nearly 50% of pupa possess extremely small heads.

### Pathological effects of glial cell-lineage expression of mutant ataxin-1

**Figure 5:**
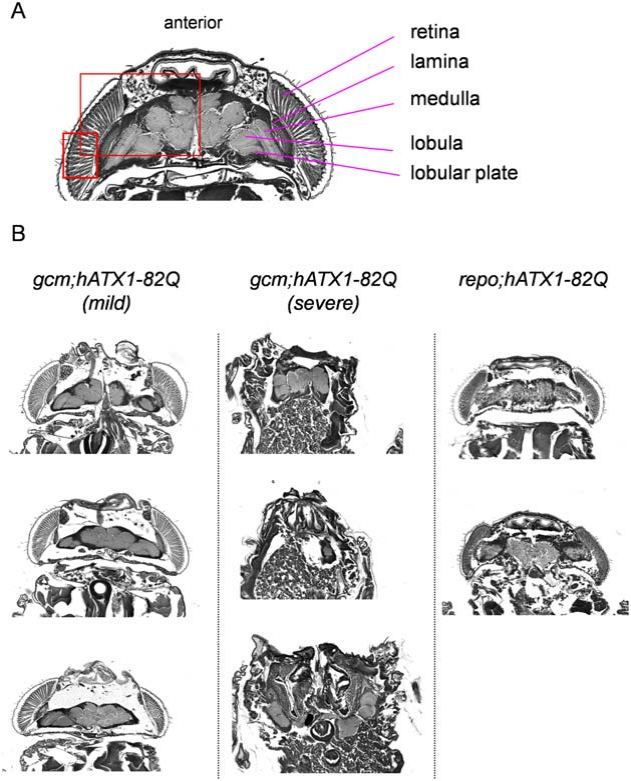
Morphological analysis of the small head phenotype in pupae expressing hAtx1-82Q in glial lineage cells. (A) The head structure of normal pupa stained by toluidine blue. The squares indicate brain and retina regions for immunohistochemistry shown in figure 6, 7. (B) Representative morphologies of the head of the *gcm; hATX1-82Q* (mild and severe cases) and *repo; hATX1-82Q* pupae are shown (toluidine blue staining).

The morphologies of pupal brains were remarkably changed both in *gcm; hAtx1-82Q* and *repo; hAtx1-82Q* pupae (Fig. 5A and B). As shown in Fig. 4C, shrinkage of the head size by *gcm-Gal4* driven expression of Atx1-82Q exhibits biphasic distribution. In mild cases, the gross structure of the central nervous system is relatively preserved, whereas the size of each brain structure became 50–60% of normal in length (Fig. 5B). In some parts of the brain, vacuolations were observed in the tissue (Fig. 5B). In severe cases, however, the brain structure was extremely destroyed and the relationship to surrounding head tissues was also distorted (Fig. 5B).

**Figure 6:**
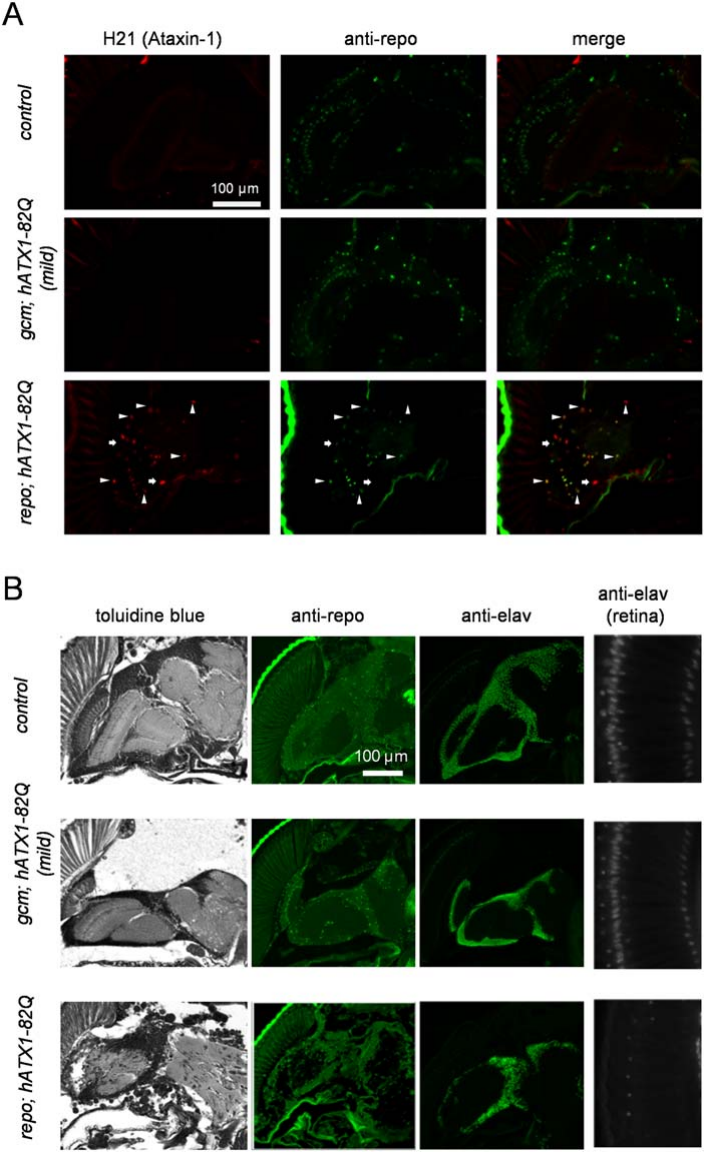
hAtx1-82Q expression in glial cell lineage induces pathological change in the central nervous system. (A) Double-staining with anti-human Atx1 (H21) and anti-repo antibodies shows co-localization of mutant hAtx1 and repo proteins in glial cells of *repo; hATX1-82Q* pupae (arrowhead). A small part cells expressed only repo or hAtx1 protein (arrow). The former would non-expressors and the latter would be ghost cells with hAtx1 inclusions. (B) Immunohistochemistry with anti-repo and anti-elav antibodies showing glial cells and neurons, respectively. The right higher magnifications show photoreceptor cells in the retina, which were remarkably reduced in *repo; hATX1-82Q* pupae.

We confirmed expression of human mutant Atx1 protein in these pupae by immunohistochemistry (Fig. 6A). In the *hATX1-82Q/+;; repo-Gal4/+ (repo; hAtx1-82Q)* flies, most glial cells co-expressed repo and ataxin-1 proteins (Fig. 6A). A small number of cells expressed only ataxn-1 probably because the cell viability was reduced (Fig. 6A, arrow heads). Glial cells expressing only repo were extremely rare (Fig. 6A arrowhead). On the other hand, in the *gcm; hAtx1-82Q* flies, surviving glial cells did not express Atx1 protein (Fig. 6A, middle panels), suggesting that neuroglioblasts/gliobalsts expressing mutant Atx1 (Fig. 1) were already selected during development and survived glial cells not expressing Atx1 were relatively healthy.

In the *repo; hAtx1-82Q* pupae, the gross structure of the brain was relatively preserved. Although the number of glial cells stained with anti-repo antibody did not change remarkably, the neuropils became coarse and the number of neurons stained with anti-elav antibody was reduced (Fig. 6B). The reduction of neurons (photoreceptor neurons) was most prominent in retina (Fig. 6B). In addition, anti-elav staining revealed that the number and density of neurons surrounding optic lobes decreased remarkable in the *gcm; hAtx1-82Q* and *repo; hAtx1-82Q* pupae.

**Figure 7:**
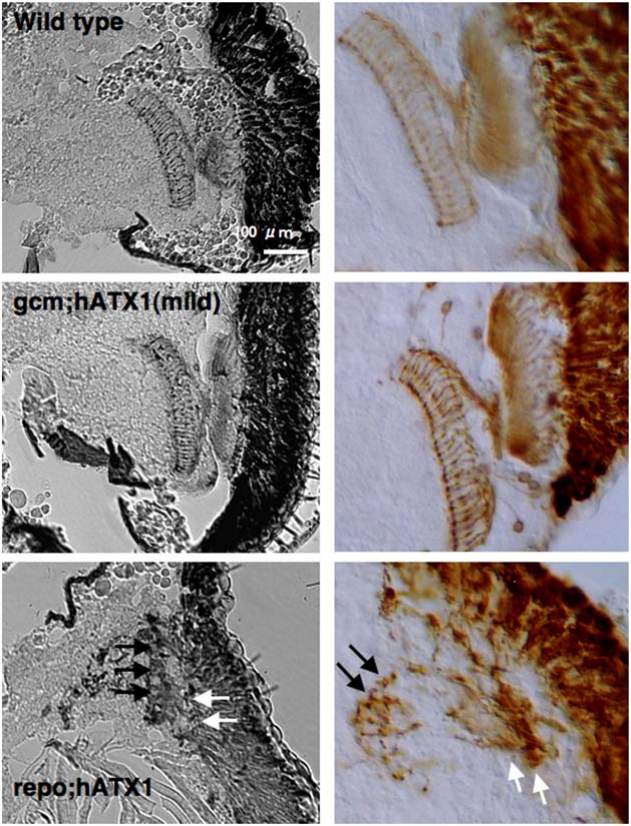
hAtx1-82Q expression in glial cell lineage induces a change in a pattern of axon. Axons from photoreceptor neurons were stained with an antibody specific for photoreceptor neurons (MAb24B10). The number of axons and their alignment were remarkably distorted especially in *repo; hATX1 82Q* pupae. Lamina and medulla were remarkably deformed (white and black arrows, respectively).

When axons from photoreceptor neurons were stained with a specific antibody (MAb24B10), the number of axons was reduced and the axonal pathway from retina to lamina and that from lamina to medulla were remarkably distorted (Fig. 7).

## Discussion

In this study, we asked generality of the glia-mediated non-cell autonomous effect and the stem/progenitor cell-mediated developmental effect in the pathology of neurodegeneration by using two different polyQ genes in Drosophila system with two different drivers for gene expression. Glial cell lineage expression of mutant polyQ proteins by the two drivers evoked profound effects on development, survival, behavior and pathology. Although the severities and the patterns of pathological/behavioral changes were different, both mutant hHtt and hAtx1 induced remarkable outcomes either through stem/progenitor cell-specific or glial cell-specific expression. These results basically support the hypothesis that developmental and non-cell autonomous (glial) effects are shared in multiple neurodegenerative disorders.

Previously, two groups performed homologous studies. Kretzschmar and colleagues expressed ataxin-3 in Drosophila neurons and glias using APPL-Gal4 and M1B-Gal4 (a Gal4-insertion in the repo gene), respectively [13]. They beautifully showed ataxin-3 aggregation in neurons and glial cells, as well as vacuolar changes of glial cells. The Birman's group analyzed effects of repo-Gal4-driven expression of Htt93Q on behaviors [14]. They nicely showed life span decrease and locomotor defects. However, these analyses were limited to a single gene. Both studies did not employ two drivers conducting glial cell lineage expression at different developmental stages. Therefore, our study added novel insights to the polyQ pathologies, including developmental effects of mutant polyQ proteins expressed in glial cell lineage stem cells and distinct effects of huntingtin and ataxin-1.

Gilal cell-specific expression of mutant hHtt by the *repo* driver leads to the lifespan shortening and the abnormal motor activities (Fig. 2A, B). Neurons, in addition to glial cells, were affected morphologically in those flies (Fig. 3B). Similarly, remarkable effects on neuronal functions and developmental defects, including neuronal loss in the retina and optic lobes, were induced by *repo*-driven exression of mutant hAtx1 (Fig. 6B). Thus, expression of the two mutant polyQ proteins in glial cells definitely induces a kind of non-cell autonomous effect on neurons.

Meanwhile, *gcm*-driven expression of mutant proteins in stem/progenitor cells of glial cell lineage induced more profound effects. In the case of hHtt, *gcm*-driven expression, either in neuroglioblasts or gliobalsts, before L1 stage during development (Fig. 1) permitted development to pupae and adult flies although the number of adult flies was reduced (Table 1). The adult flies survived developmental stress have short lifespan (Fig. 2A), suggesting that impairment of stem/progenitor cells induces certain delayed effect(s) on the nervous tissue. Our result is consistent with the findings by the other group that hAtx1 interaction with ROR-α induced delayed effect in the model mice [7].

The two disease genes driven by the same driver induced different effects. Mutant hAtx1 expression did not permit development to adult flies in either driver (Fig. 4A). The severities of phenotypes by *repo* and *gcm* drivers were almost similar. On the other hand, mutant hHtt expression driven by *repo* and *gcm* drivers permitted development to adult flies. The survival of adult flies was shorter in *gcm*-driven expression than in *repo*-driven expression (Fig. 2A).

The discrepancy in phenotypic severities between hHtt and hAtx1 might come from a higher conservation of Atx1 and its interacting molecules in Drosophila. Drosophila Htt is remarkably different from human Htt, while Atx1 possesses 44% of homology in the AXH domain between Drosophila and human [15]. The AXH domain mediates neurotoxicity of hAtx1 through interaction with Gfi-1/Senseless, which is conserved in human and Drosophila [15]. Capicua, a critical interacting molecule to hAtx1 that mediates physiological functions of hAtx1 through binding to phosphorylated Ser776 proximal to the AXH domain [16], [17], is also highly conserved between human and Drosophila. Therefore, not only gain of abnormal function but also loss of physiological function might be conserved in Drosophila models similarly to human SCA1 pathology. Another possibility is that exon-1 of mutant hHtt was expressed in our flies. The partial molecule might be less toxic than the full-length hAtx1. On the other hand, as our fly models are expressing the partial molecule, loss of physiological function of hHtt [18] is not highly plausible in our case.

Diversity in the phenotypes of the *gcm*-driven hAtx1 expressing flies is an open question. As the neuronal pathways are relatively preserved in the mild cases of *gcm; hAtx1-82Q* flies and survivors show relatively normal glial cells without mutant hAtx1, we might be able to assume that the stem/progenitor cell survival against mutant hAtx1 is stochastically regulated by a certain gene. As the ratio between severe and mild cases was nearly 1∶1, such a single gene might regulate the phenotype diversity in *gcm; hAtx1-82Q* flies. It would be interesting to identify the gene through genetic screening. Although we mainly analyzed adult flies in the case of mutant hHtt expression, the birth rate of the hHtt expressers with the *gcm-Gal4* driver was remarkably lower than that with the *repo-Gal4* driver (Table 1), suggesting that embryonic death also occurs in mutant hHtt expression. Identification of such a modifier gene might provide us some hints for therapeutics development.

In conclusion, our fly models expressing mutant hHtt and hAtx1 in glial cells or glial lineage stem/progenitor cells have extended the non-cell autonomous pathology hypothesis as well as the developmental effect hypothesis to multiple polyQ diseases. The two pathologies might be generally shared in neurodegeneration.

## Materials and Methods

### Fly stocks and rearing conditions

All flies were raised on a corn-meal medium without propionic acid and were maintained at 25°C and 60% humidity under a 12∶12 hr light-dark cycle. pUAST-hHtt 103Q plasmid was generated by subcloning human *HD* exon 1 cDNA digested from pTL1HA3-HD90Q [19] with EcoRI and NotI, into pUAST vector. During the subcloning, CAG repeats were expanded. Transgenic flies of mutant Atx1 containing human full-length Atx1 with 82Q (*y^1^w^1118^* UAS:ATX1 82Q) were described previously [20], [21]. *w^1118^*; *P{GAL4}repo/TM3*, *Sb^1^* and *gcm-Gal4/Gla* flies were obtained from the Bloomington *Drosophila* stock center. Canton-S strain was used as the wild-type control in this study.

### Calculation of toxicity of hAtx1 during larval and pupa stage

Males of *gcm-Gal4* and *repo-Gal4* driver flies which were balanced by GFP-balancers, *gcm-Gal4/CyO*, *GFP* and *repo-Gal4/TM3*, *GFP Sb* were crossed with *UAS-hATX1 82Q* homozygous virgin females respectively. We randomly picked up F1 pupa or larvae and checked their GFP under a stereo fluorescent microscope (LEICA, MZFLIII). Ratio of GFP (−)/GFP (+) larva or pupa were calculated, if expression of hAtx1-82Q at glial cells is not toxic the ratio should be 1.

### Survival assay

For measurement of lifespan, about 25 virgin females were reared in a food vial and transferred to fresh food vials every 2 or 3 days. Numbers of dead flies were counted every 1–2 days.

### Spontaneous activity assay

For spontaneous activity analysis virgin males were placed individually in glass tubes with one end filled with medium and another end cotton. Their motion is detected and counted by infrared light beam breaks every 30 min using a Drosophila Activity Monitoring System (Trikinetics, Waltham, MA). To calculate synchronized zeitgeber rhythm the flies were kept under 12 hour light-12 hour dark cycles (LD) for 10 days.

### Negative geotaxis assay

Individual female fly was transferred to a test column (150 mm in length and 25 mm in diameter) lined with nylon mesh. To evaluate climbing ability of flies, we used the startle-induced climbing assay, which had been developed by Bainton et al [22]. The bottom of test column was tapped against a soft surface of the bench top to drop flies to the bottom after they had been placed there for 20 sec. Although almost all flies in column were dropped to the bottom by one tap, we used three taps to drop all flies to the bottom. The time that flies reach 50 mm from the bottom were counted. All flies tested reached at 50 mm within 10 sec.

### Paraffin sections and Immunohistochemistry

Proboscis were removed from dissected adult female fly heads or mature pupae and were fixed in carnoy's solution (ethanol∶ chloroform∶ acetate = 6∶3∶1) for 3 hours at 4°C, dehydrate in serial dilutions of ethanol and embedded in paraffin (pathoprep546, m.p. 54∼56°C, Wako). The paraffin blocks were cut into 6 µm horizontal (for pupa) or frontal (for adult heads) sections. After re-hydration, the sections were stained with anti-huntingtin N-18 antibody (Santa Cruz, diluted 1∶100), Ataxin-1 H21 antibody (Santa Cruz, diluted 1∶100), anti-elav or anti-repo (developmental studies hybridoma bank, Iowa University, diluted 1∶10 and 1∶50), and with Alexa488-conjugated secondary antibody (Jackson). Cy3-conjugated secondary antibody (Jackson) was used for double staining. The sections for N-18, H21 and anti-repo antibodies were treated with microwave before staining.

### Frozen sections and Immunohistochemistry

Mature pupae were fixed in 4% paraformaldehyde in PBS for 1.5 hours followed by successive incubations in 5% and 10% sucrose for 30 minutes, 15% and 20% sucrose for 1 hour, and 30% sucrose overnight at 4 degree. All sucrose solutions were in PBS. After the heads or mature pupae were frozen in dry ice/ n-hexane, 10 µm frontal sections were cut with a cryostat microtome. They were then stained with anti-chaoptin antibody (24B10) (developmental studies hybridoma bank, Iowa University, diluted 1∶200), Alexa488-conjugated secondary antibody (Jackson).

### Toluidine Blue Staining

Toluidine blue staining of adult heads or mature pupae was performed as we described previously [21], [23]. Briefly, paraffin sections (6 µm) were stained with 0.5% toluidine blue (Merck) plus 0.5% Borax after re-hydration.
